# Calcineurin-dependent 20E signaling pathway enhances attacin expression to prime the immunity of *Helicoverpa armigera* against *Bacillus thuringiensis* Cry1Ac toxin

**DOI:** 10.1371/journal.ppat.1014503

**Published:** 2026-08-07

**Authors:** Xue Yao, Pin Li, Yuge Zhao, Neil Crickmore, Gemei Liang, Mengfang Du, Xianchun Li, Xinming Yin, Shiheng An, Jizhen Wei

**Affiliations:** 1 College of Plant Protection/State key Laboratory of High-Efficiency Production of Wheat-Maize Double Cropping, Henan Agricultural University, Zhengzhou, China; 2 School of Life Sciences, Henan University of Animal Husbandry and Economy, Zhengzhou, China; 3 School of Life Sciences, University of Sussex, Brighton, United Kingdom; 4 State key Laboratory for Biology of Plant Diseases and Insect Pests, Institute of Plant Protection, Chinese Academy of Agricultural Science, Beijing, PR China; 5 School of Agricultural Sciences, Zhengzhou University, Zhengzhou, China; National Institutes of Health, UNITED STATES OF AMERICA

## Abstract

Enhancing the insecticidal efficacy of *Bacillus thuringiensis* (Bt) toxins by suppressing the immune responses of target insects can simultaneously augment the effectiveness of Bt-based products and address the growing issue of resistance. Our previous studies found that the inhibition of calcineurin (CaN) activity can enhance the toxicity of Cry1Ac, a Bt toxin, against *Helicoverpa armige*ra, and that CaN regulates the expression of antimicrobial peptides (AMPs) in response to external pathogen invasion through the transcription factor Relish in *H. armigera*. Our objective was to investigate the regulatory relationship between AMPs expression and susceptibility to Cry1Ac. Our findings demonstrated that the transcription factors *Relish* and *Dorsal* mediate the CaN-induced upregulation of attacin expression, while attacin itself interacts with Cry1Ac to reduce its toxicity against *H. armigera*. Furthermore, Cry1Ac treatment results in elevated levels of 20E in *H. armigera*, partly triggering an increase in intracellular Ca^2+^ concentration, which in turn activates CaN. These findings contribute to the robust body of evidence that insects can elicit immune defenses as an adaptive mechanism against Bt toxins, and also offer a potential strategy for improving pest control effectiveness in agricultural settings.

## Author summary

Cry1Ac is an insecticidal protein produced by the bacterium *Bacillus thuringiensis*, is widely used to protect crops from pests. Immune responses regulated by calcineurin (CaN) represent a crucial pathway for insects to defend against external substances. Our previous study found that inhibiting CaN activity increases the susceptibility of *H. armigera* to Cry1Ac toxicity. Present study investigates the underlying mechanism and reveals a self-reinforcing immune defense pathway triggered by Cry1Ac exposure in *H. armigera*: Cry1Ac activates the 20E/Ca² ⁺ /CaN signaling cascade, whereby CaN promotes the expression of attacin by dephosphorylating the transcription factors Relish and Dorsal. Overexpressed attacin attenuates Cry1Ac toxicity by binding to the Cry1Ac protein. Interestingly, this immune defense mechanism is also operative in a *H. armigera* strain that has acquired resistance to Cry1Ac. In conclusion, our findings reveal a previously unknown immune adaptation to Bt toxins in insects and offer new targets for improving the effectiveness of biological pesticides and slowing the development of resistance.

## 1. Introduction

The insect pathogen *Bacillus thuringiensis* (Bt) stands out as the most reliable and effective biological control agent to date, largely due to its remarkable insecticidal activity, which is primarily attributed to δ-endotoxin proteins. However, beyond the development of insect resistance to Bt, the intrinsic immune responses of insects and nematodes also play a critical role in attenuating the virulence of Bt pesticidal proteins [1–5]. In particular the phenomenon known as “immune priming” in insects, whereby insects can extend the activation of their immune responses and transmit this enhanced immunity to their offsprings [6,7]. Inhibiting the immune response of invertebrates is an important potential strategy to enhance the toxicity of Bt and manage the development of resistance to Bt [1,8]. Calcineurin (CaN) plays a pivotal role as an immune-regulated gene in numerous immune pathways, and we discovered that inhibiting CaN activity significantly enhanced the insecticidal activities of Bt proteins [9,10]. For instance, the application of Cyclosporin A, an inhibitor of CaN, increased efficacy of Bt proteins against Cry1Ac-resistant cotton bollworms [11], although the precise mechanisms underlying this enhancement remain to be fully elucidated.

Research on insects has demonstrated that CaN, a Ca^2+^-dependent phosphatase, promotes innate immunity by regulating the expression of antimicrobial peptides (AMPs) through the Toll and Imd signaling pathways [12]. Serving as an immune regulator, CaN interacts with the transcription factor Relish to regulate the expression of AMPs such as gloverin, cecropin D, and attacin in *H. armigera* [13]. Many studies have reported that various insect species show induced expression of AMPs such as gloverin, moricin, lebocin, attacin, cecropins, and cobatoxin A in response to Bt intoxication [14–19]. These AMPs are small peptides that are widely present in nature and play a crucial role in the innate immune response of various organisms, with their biosynthesis mediated by the Toll, Imd, and JAK/STAT signaling pathways [1]. Despite this, the mechanisms by which these over-expressed AMPs influence the toxicities of the Bt pesticidal proteins are not well understood.

CaN, a member of the protein phosphatase 2B family, depends on Ca^2+^ signaling for its activity to regulate gene transcription [20,21]. Furthermore, research findings also demonstrate that Bt treatment increases intracellular Ca^2+^ concentration [22–24]. More importantly, this rise in Ca^2+^ levels did not trigger cellular death in response to Cry1A toxins [25]. To understand the mechanism by which CaN inhibition modulates susceptibility to Bt, it is essential to investigate whether the elevation in Ca^2+^ concentration induced by Cry1Ac activates CaN and subsequently influences the expression of AMP genes.

Studies have shown that insect ecdysone (20-hydroxyecdysone, 20E) triggers Ca^2+^ release and phosphorylation of stromal interacting molecule 1 (STIM1) at Ser-485 via the G protein–coupled receptors (GPCRs)/PLC/IP3R/PKC signaling pathways, leading to STIM1 aggregation. The aggregated STIM1 subsequently translocates toward the endoplasmic reticulum-plasma membrane (ER-PM) junction and interacts with orai1 on the plasma membrane, thereby facilitating Ca^2+^ entry [26]. Moreover, 20E can modulate immune responses to activate the expression of antimicrobial peptide genes [27]. Interestingly, increased 20E titers were observed in Cry1Ac-resistant *Plutella xylostella* [28,29], suggesting a potential involvement of this hormone in mediating immune defenses against Cry1Ac in this species.

We propose that Cry1Ac treatment leads to an elevation in 20E titer, which subsequently causes an increase in intracellular Ca^2+^ concentration, this elevation activates CaN, subsequently inducing the expression of AMPs via the action of transcription factors. The current investigation aims to test this proposed mechanism and explore the functions of AMPs in Bt toxicity. Elucidating the immune signaling pathways activated by Bt exposure, and identifying the key factors in their immune response, will provide insights into managing insect resistance to Bt and contribute to the development of more effective Bt-based pest control strategies.

## 2. Result

### 2.1. Cry1Ac induced CaN activity

**Fig 1:**
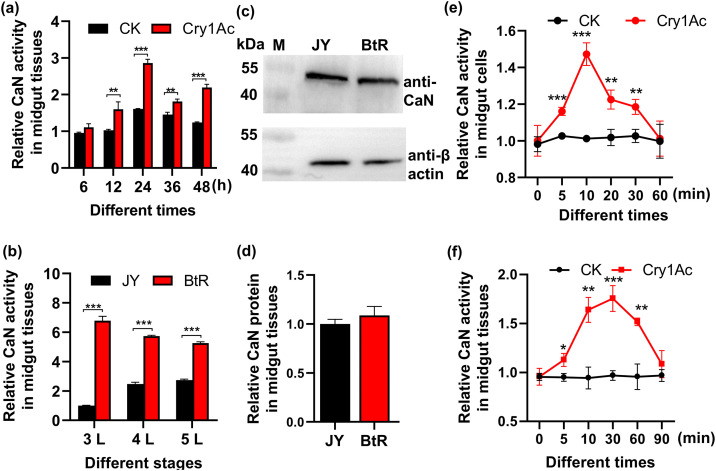
CaN activity in Cry1Ac-induced *H. armigera* larvae, *H. zea* MG cells and *H. armigera* midgut tissues as well as in Cry1Ac-resistant *H. armigera* larvae. **(a)** Alterations in CaN activity observed in susceptible larvae following ingestion of Cry1Ac protein (n = 4 - 5). **(b)** CaN activity in midgut tissues of resistant and susceptible larvae at various developmental stages in the absence of Cry1Ac exposure (3rd: n = 10 - 15, 4th: n = 6 - 10, 5th: n = 6 - 10). **(c)** CaN protein levels in midgut tissues of 5th instar larvae from resistant (BtR) and susceptible (JY) strains. **(d)** Densitometry analysis of **(c)**. **(e)** The effect of activated Cry1Ac protein on CaN activity in MG cells. **(f)** The effect of activated Cry1Ac protein on CaN activity in *H. armigera* midgut tissues (n = 5 - 6). CK, control group. Values shown are means and standard errors. Significant differences among the different treatments are indicated by asterisks (*P < 0.05, **P < 0.01, ***P < 0.001 based on Student *t*-tests, DPS7.05).

We had previously shown that CaN functions as an important immune regulator in *H. armigera* and that its inhibition led to reduced expression of antimicrobial peptides including attacin [13]. Since CaN has also been implicated in modulating the response of *H. armigera* to Bt toxins [9,30], we aimed to determine whether CaN-induced expression of AMPs contributed to the defense of this insect against the Cry1Ac protein. To assess this, the activity of CaN was measured in fifth instar larvae after treatment with sublethal doses of Cry1Ac (LC_30_). The results revealed a significant increase in CaN enzyme activity in Cry1Ac-fed larvae (Fig 1a; 6 h: *t* = 2.573, *P* = 0.0618; 12 h: *t* = 4.8177, *P* = 0.0085, 24 h: *t* = 20.8842, *P <* 0.001; 36 h: *t* = 6.5293, *P* = 0.0028, 48 h: *t* = 18.5668, *P <* 0.001, respec*t*ively). A higher level of CaN activity was also observed in the midgut of resistant larvae, compared to the susceptible insects, in the absence of Cry1Ac treatment (Fig 1b;3 L: *t* = 33.9790, *P <* 0.001, 4 L: *t* = 41.5678, *P <* 0.001, 5 L: *t* = 43.6659, *P <* 0.001, respectively). Al*t*hough a greater CaN ac*t*ivity was observed in the resistant larvae, there was no significant difference in the amount of protein produced (Fig 1c, d; *t* = 1.8719, *P* = 0.0981), suggesting that post-*t*ranslational modifications may account for the increased activity. As with *H. armigera* larvae we also found that Cry1Ac treatment caused a significant, but transient, rise in the enzyme activity of CaN in both *H. zea* midgut cells (Fig 1e; 0 min: *t* = 0.3288, *P* = 0.7588, 5 min: *t* = 9.8006, *P* = 0.0006, 10 min: *t* = 12.2851, *P* = 0.0003, 20 min: *t* = 5.2638, *P* = 0.0062, 30 min: *t* = 5.0254, *P* = 0.0074, 60 min: *t* = 0.1856, *P* = 0.8618, respec*t*ively) and *H. armigera* midgu*t t*issues (Fig 1f; 0 min: *t* = 0.0308, *P* = 0.9769, 5 min: *t* = 3.9418, *P* = 0.0169, 10 min: *t* = 7.1079, *P* = 0.0021, 30 min: *t* = 9.6357, *P* = 0.0006, 60 min: *t* = 7.1030, *P* = 0.0021, 90 min: *t* = 1.3321, *P* = 0.2536, respec*t*ively).

### 2.2. Cry1Ac induced attacin expression and was bound by induced attacin

**Fig 2:**
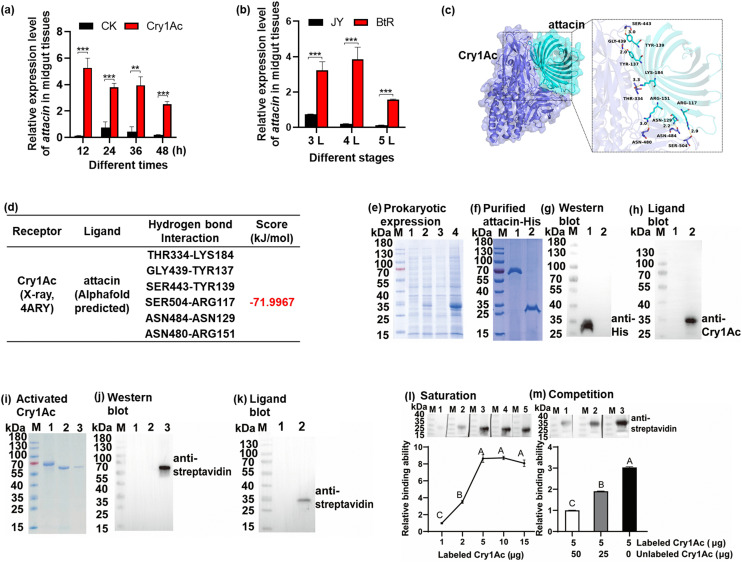
Cry1Ac inducibility of attacin and its binding ability to Cry1Ac. **(a)** Transcription levels of the attacin gene in midgut tissues of larvae from the susceptible strain following ingestion of Cry1Ac. CK, control group (n = 4 - 5). **(b)** Attacin gene expression levels in midgut tissues of larvae derived from resistant (BtR) and susceptible (JY) strains (3rd: n = 10 - 15, 4th: n = 6 - 10, 5th: n = 6 - 10). **(c)** Schematic diagram of molecular docking between the Cry1Ac protein and attacin. **(d)** Molecular docking sites and binding score. **(e)** SDS-PAGE analysis of attacin protein expression induced by IPTG from the pET30a-attacin-His plasmid. M: protein marker; 1: no induction; 2: proteins induced by IPTG; 3: supernatant fraction; 4: pellet fraction. **(f)** SDS-PAGE analysis of purified attacin. M: protein marker; 1: BSA; 2: purified attacin-His. **(g)** Validation of attacin protein expression via western blot analysis. M: protein marker; 1: induced proteins; 2: non-induction control. **(h)** Binding of activated Cry1Ac protein to immobilized attacin. M: protein marker; 1: BSA; 2: purified attacin-His. **(i)** SDS-PAGE analysis of activated Cry1Ac protein labeled with biotin. M: protein marker; 1: BSA protein; 2: unlabeled Cry1Ac; 3: labeled Cry1Ac. **(j)** Western blot analysis confirming the successful biotin labeling of activated Cry1Ac. M: protein marker; 1: BSA protein; 2: unlabeled Cry1Ac; 3: labeled Cry1Ac. **(k)** Interaction between labelled Cry1Ac and attacin confirmed with HRP streptavidin. M: protein marker; 1: BSA; 2: purified attacin. **(l)** Saturation binding assays between attacin and activated Cry1Ac. M: protein marker; 1/2/3/4/5: purified attacin-His. **(m)** Homologous competition between labeled Cry1Ac and unlabeled Cry1Ac to attacin. M: protein marker; 1/2/3/: purified attacin-His. The quantitative determination of the relative intensity was conducted using Image **J.** Different letters indicate significant differences at the *P <* 0.01 level by Tukey test. Significant differences among the different treatments are indicated by asterisks (**P < 0.01, ***P < 0.001 based on Student *t*-tests, DPS7.05).

Following the finding that exposure to Cry1Ac induce an increase in CaN activity, we sought to determine whether such exposure similarly enhances the expression of AMPs. Indeed, upon treating the susceptible strain with Cry1Ac, a significant upregulation of the AMP attacin was observed (Fig 2a; 12 h: *t* = 17.2945, *P* *<* 0.001, 24 h: *t* = 10.2419, *P* *<* 0.001, 36 h: *t* = 8.1570, *P* = 0.0012, 48 h: *t* = 25.0421, *P <* 0.001, respec*t*ively). Moreover, larvae from *t*he Cry1Ac-resistant strain exhibited elevated attacin levels as well (Fig 2b; 3 L: *t* = 8.8925, *P* *<* 0.001, 4 L: *t* = 9.3007, *P* *<* 0.001, 5 L: *t* = 108.9519, *P* *<* 0.001, respec*t*ively). A*tt*acins are glycine-rich antimicrobial pro*t*eins that are predominantly active against gram-negative bacteria and act via interacting with the cell membrane and inducing cell lysis [31]. Their production in response to a bacterial pore-forming toxin may serve either as a defense mechanism against the producing bacterium or the virulence factor themselves. To investigate the latter possibility, we looked at a potential interaction between attacin and Cry1Ac utilizing a molecular docking approach. The analysis revealed multiple hydrogen bonds between residues of attacin and Cry1Ac (Fig 2c-2d), resulting in a binding energy of -71.9967 kJ/mol, indicative of a stable interaction (Fig 2d). To physically confirm this potential interaction, a His-tagged attacin protein was expressed. SDS-PAGE analysis revealed a distinct band of within the expected molecular weight range of 25–35 kDa (Fig 2e). Subsequent affinity purification and refolding, yielded a pure sample (Fig 2f) and its identity confirmed by western blotting using anti-His tag antibodies (Fig 2g). A far-western blot assay was then conducted, wherein a membrane immobilizing attacin was incubated with Cry1Ac and probed with anti-Cry1Ac antibodies, revealing a definitive binding between attacin and Cry1Ac (Fig 2h). To further confirm this interaction, a homologous competition experiment was conducted. Cry1Ac was first labelled with biotin (Fig 2i, 2j) and demonstrated to bind attacin in a dose-dependent manner via far-western blot (Fig 2k, 2l; *F* = 426.649, *P* *<* 0.001). Furthermore, unlabeled Cry1Ac competed effectively with the biotinylated toxin for attacin binding (Fig 2m; *F* = 6257.561, *P* *<* 0.001), confirming the specificity of the attacin-Cry1Ac interaction.

### 2.3. Attacin attenuated the activity of Cry1Ac to *H. armigera* larvae and Sf9 cells

**Fig 3:**
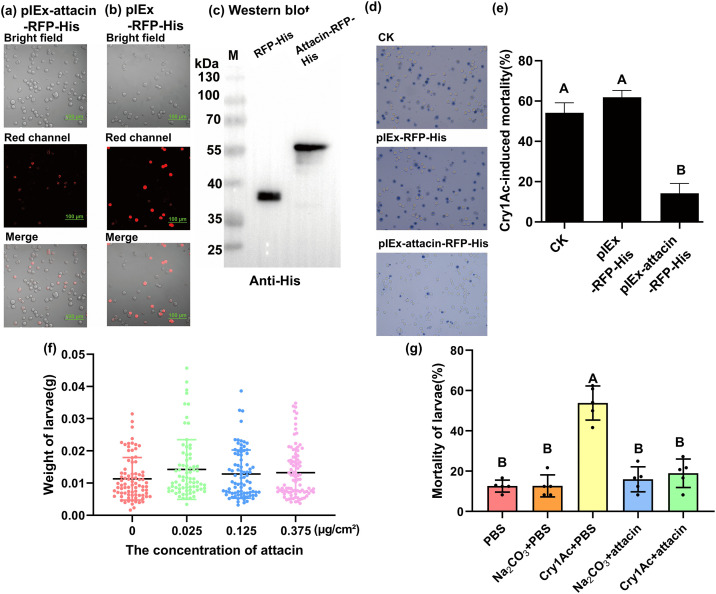
The effect of over-expressing attacin in Sf9 cells and of feeding attacin to larvae on their susceptibility to Cry1Ac. **(a)** Sf9 cells transfected with pIEx-attacin-RFP-His **(b)** Sf9 cells transfected with pIEx-RFP. **(c)** Western blot analysis of expressed attacin protein. M: marker. **(d)** Cell death in transfected cells treated with activated Cry1Ac. **(e)** Graphical representation of the data from **(d)**. **(f)** The effect of feeding different concentrations (0, 0.025, 0.125 and 0.375 μg/cm²) of attacin protein in PBS on the body weight of newly hatched susceptible larvae (n = 70 - 90). **(g)** The effect of feeding a combination of 0.025 μg/cm² Cry1Ac in Na_2_CO_3_ and 0.375 μg/cm² attacin in PBS on the mortality of newly hatched larvae from the susceptible strain (n = 25 - 30). Different letters indicate significant differences at *P <* 0.01 level by Tukey test.

The data presented above suggest a physical association between Cry1Ac and attacin, however, evidence for a physiological interaction remained to be established. To achieve this *H. armigera* attacin was expressed in Sf9 cells. Two distinct plasmids were transfected into this cell line, one plasmid (pIEx-RFP-His) encoded only the red fluorescent protein while the other (pIEx-attacin-RFP-His) expressed a fusion protein combining attacin with RFP-His. Transfection efficiency was verified by fluorescence microscopy 24 hours post-transfection (Fig 3a, 3b), and attacin expression was confirmed via western blot analysis at 48 hours (Fig 3c) Subsequent treatment of the transfected cells with activated Cry1Ac protein showed that cells expressing attacin exhibited a significantly lower mortality rate (Fig 3d, 3e; *F* = 97.029, *P* *<* 0.001). When purified attacin was fed to newly hatched larvae, no significant change in larval weight was observed (Fig 3f; *F* = 1.444, *P* = 0.2313). However, feeding larvae a mixture containing both attacin and Cry1Ac effectively counteracted the toxic impact caused by Cry1Ac alone (Fig 3g; *F* = 38.698, *P* *<* 0.001). The above results indicate that attacin can interact with Cry1Ac and alter its toxic effect.

### 2.4. Cry1Ac induction of attacin was mediated by Relish transcription factor

**Fig 4:**
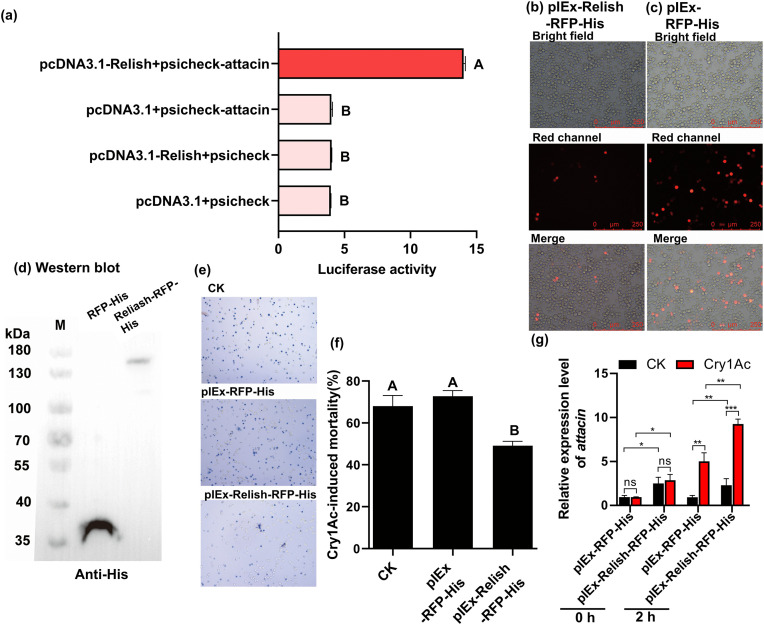
Relish regulates the expression of attacin and affects the toxicity of Cry1Ac to MG cells. **(a)** The effects of the transcription factor Relish on attacin promoter activity. **(b)** Transfection of pIEx-Relish-RFP-His in MG cells. **(c)** Transfection of pIEx-RFP-His in MG cells. **(d)** Western blot analysis of Relish protein expression. M: marker. **(e)** Cell death in transfected cells treated with activated Cry1Ac protein. **(f)** Graphical representation of the data from **(e)** Cell death in transfected cells treated with activated Cry1Ac. **(g)** Expression of the attacin gene in Relish overexpressed MG cells in response to Cry1Ac. CK, control group. The error bars indicated the mean ± SD. Different letters indicate significant differences at *P <* 0.01 level by Tukey test. Significant differences of attacin expression among the different treatments are indicated by asterisks (*P < 0.05, **P < 0.01, ***P < 0.001 based on Student *t*-tests, DPS7.05).

Our previous study indicated that CaN bound to Relish to regulate the expression of attacin [13]. To further confirm this finding, the genomic sequence of attacin (GenBank: AY948540.1) was retrieved from NCBI, and its promoter region was inserted into upstream of a luciferase reporter gene. Co-expression of Relish led to an increase in promoter-driven transcription (Fig 4a; *F* = 12663.239, *P* *<* 0.001) indicating a direct interaction between the transcription factor and the attacin promoter. To investigate the functional consequences of Relish expression the protein was expressed as an RFP fusion in MG cells, successful expression was confirmed via fluorescence microscopy and western blot analyses (Fig 4b, 4c, 4d). When cells expressing Relish were treated with Cry1Ac, mortality was reduced compared to the control groups (Fig 4e, 4f; *F* = 37.475, *P* *<* 0.001). Moreover, Relish-expressing cells showed elevated attacin levels, which were further enhanced following treatment with a sublethal Cry1Ac dose (Fig 4g). Collectively, these results support the role of Relish in upregulating attacin expression, thereby conferring cellular protection against Cry1Ac toxicity.

### 2.5. Cry1Ac-induced CaN activity was responsible for the Cry1Ac-triggered transient dephosphorylation of the Dorsal transcription factor

**Fig 5:**
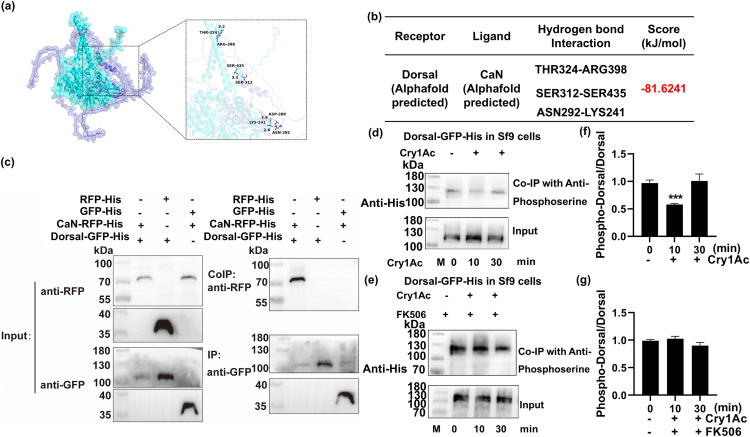
Binding interactions between CaN and Dorsal, and the effects of FK506 and Cry1Ac on the dephosphorylation of Dorsal. **(a)** Schematic diagram of molecular docking between CaN and Dorsal. **(b)** Molecular docking sites and scores. **(c)** Co-IP experiment between Dorsal and CaN. The input blots indicate whether the transfected plasmid has successfully expressed the protein in the cells. IP indicates which GFP-containing proteins have been pulled down by the anti-GFP antibody. Co-IP indicates which RFP-containing proteins have been pulled down alongside the GFP ones. **(d)** Co-IP experiment indicating how much Dorsal protein has been pulled down by an anti-phosphoserine antibody following Cry1Ac treatment. **(e)** As above but in the presence of FK506 **(f)** Densitometry analysis of the data from **(d)**. **(g)** Densitometry analysis of the data from **(e)**. Significant differences among the different treatments are indicated by asterisks (***P < 0.001 based on Student’s *t*-*t*ests, DPS7.05).

It had previously been reported that Dorsal is another transcription factor that CaN interacts with to regulate AMP expression [12]. The hydrogen bond interaction between Dorsal (G3LF42) and CaN (A0A0U2JFS6) was investigated by molecular modelling, revealing a potentially strong interaction (Fig 5a, 5b). To confirm this interaction a co-immunoprecipitation assay was performed using cells expressing fluorescently tagged Dorsal and CaN. The results (Fig 5c) demonstrated that precipitating Dorsal also co-precipitated CaN, confirming a direct physical association between these proteins. Since the activity of Dorsal was known to be affected by its phosphorylation status, we investigated any relationship between the phosphorylation status of Dorsal and the response to Bt intoxication. Cells expressing Dorsal-GFP-His were exposed to sublethal doses of activated Cry1Ac protein which resulted in a transient decrease in the phosphorylation level of Dorsal (Fig 5d). Importantly, this effect was abolished upon treatment with the CaN inhibitor FK506 (Fig 5e).

### 2.6. Cry1Ac induction of attacin was also mediated by Dorsal

**Fig 6:**
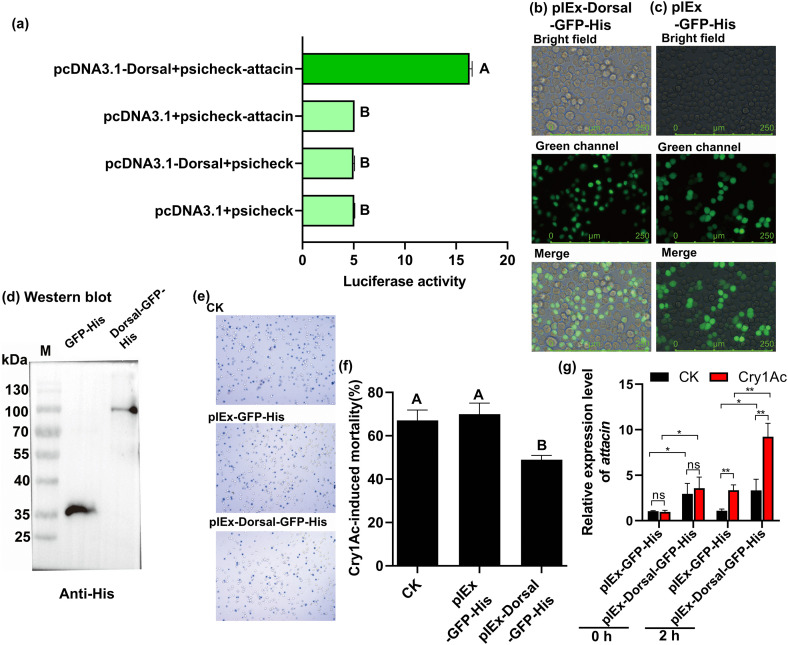
Dorsal regulates the expression of attacin and affects the toxicity of Cry1Ac in MG cells. **(a)** The effects of the transcription factor dorsal on attacin promoter activity. **(b)** Transfection of pIEx-Dorsal-GFP-His in MG cells. **(c)** Transfection of pIEx-GFP-His in MG cells. **(d)** Western blot analysis confirming the successful expression of dorsal protein. M: marker **(e)** Cell death in transfected cells treated with activated Cry1Ac protein. **(f)** Cell mortality in transfected cells treated with activated Cry1Ac protein. **(g)** The effects of Cry1Ac-active proteins on attacin transcript levels. CK, control group. The error bars indicated the mean ± SD. Different letters indicate significant differences at the *P <* 0.01 level by Tukey test. Significant differences of attacin expression among the different treatments are indicated by asterisks (*P < 0.05, **P < 0.01, ***P < 0.001 based on Student *t*-tests, DPS7.05).

The data presented above align with the hypothesis that Dorsalis involved in the CaN-mediated regulation of attacin gene expression. To demonstrate a direct link Dorsal was co-expressed in a cell containing an attacin promoter-luciferase fusion. As illustrated in Fig 6a (*F* = 6038.404, *P* *<* 0.001), the transcription level was significantly increased in the presence of Dorsal. Green fluorescence observed under fluorescence microscopy confirmed the expression of Dorsal protein in the cells (Fig 6b, 6c). The successful transfection with the pIEx-Dorsal-GFP-His plasmid was verified through analysis of band position and molecular size (Fig 6d). Treating MG cells transfected pIEx-Dorsal-GFP-His plasmids with 30 μg/mL Cry1Ac activated protein significantly reduced the mortality rate (Fig 6e, 6f; *F* = 35.822, *P* *<* 0.001), suggesting that overexpression of the Dorsal protein can attenuate the toxicity of Cry1Ac activated protein towards MG cells. Furthermore, cells expressing Dorsal and treated with a sublethal dose of Cry1Ac demonstrated a significant increase in attacin gene expression at 2 hours post-treatment (Fig 6g).

### 2.7. Cry1Ac-induced enhancement of CaN activity was mediated by intracellular Ca^2+^ burst caused by Cry1Ac

**Fig 7:**
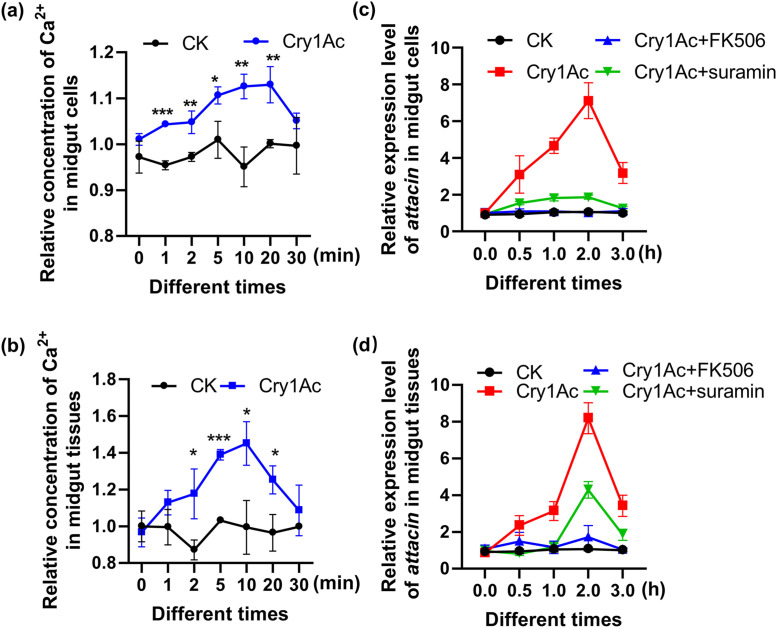
Cry1Ac affected Ca^2+^ concentration and transcription level of attacin gene. **(a)** The effect of Cry1Ac protein on intracellular Ca^2+^ concentration in MG cells. **(b)** The effect of Cry1Ac protein on intracellular Ca^2+^ concentration in midgut tissues (n = 5 - 6). **(c)** Transcription level changes of attacin gene in MG cells in response to Cry1Ac, FK506 and suramin. Significant differences among the different treatments are indicated in S1 Table. **(d)** Transcription level changes of the attacin gene in midgut tissues in response to Cry1Ac, FK506, and suramin (n = 5 - 6). CK, control group. Significant differences among the different treatments are indicated in S2 Table. Error bars indicated the mean ± SD of three independent biological experiments and three technical repetitions. Significant differences among the different treatments are indicated by asterisks (*P < 0.01, **P < 0.01, ***P < 0.001 based on Student’s *t*-tests, DPS7.05).

Since CaN is known to be activated by Ca²⁺ [20,21]. We aimed to determine whether the Cry1Ac-induced activation of CaN was mediated by Ca² ⁺ . Treatment of *H. zea* MG cells with a sublethal concentration of Cry1Ac resulted in a significant increase in Ca² ⁺ concentration (Fig 7a;0 min: *t* = 1.8049, *P* = 0.1454, 1 min: *t* = 14.5518, *P* *<* 0.001, 2 min: *t* = 4.9077, *P* = 0.0080, 5 min: *t* = 3.7310, *P* = 0.0203, 10 min: *t* = 5.9527, *P* = 0.0040, 20 min: *t* = 5.4879, *P* = 0.0054, 30 min: *t* = 1.4754, *P* = 0.2141, respec*t*ively). similar increase was observed in *H. armigera* midgut tissues (Fig 7b; 0 min: *t* = 0.4974, *P* = 0.6450, 1 min: *t* = 1.9393, *P* = 0.1245, 2 min: *t* = 3.6148, *P* = 0.0225, 5 min: *t* = 21.2244, *P* *<* 0.001, 10 min: *t* = 4.1732, *P* = 0.0140, 20 min: *t* = 3.9740, *P* = 0.0165, 30 min: *t* = 1.1059, *P* = 0.3308, respec*t*ively). Fur*t*hermore, the Cry1Ac-induced expression of attacin in both cell (Fig 7c) and tissue (Fig 7d) samples was reduced both in the presence of the CaN inhibitor FK506 and the calcium channel antagonist suramin. These findings support a model whereby Cry1Ac stimulates CaN activation through an increase in cellular calcium concentration.

### 2.8. Cry1Ac treatment resulted in an elevation of 20E titer, subsequently modulating Ca^2+^ concentrations and influencing CaN activity

**Fig 8:**
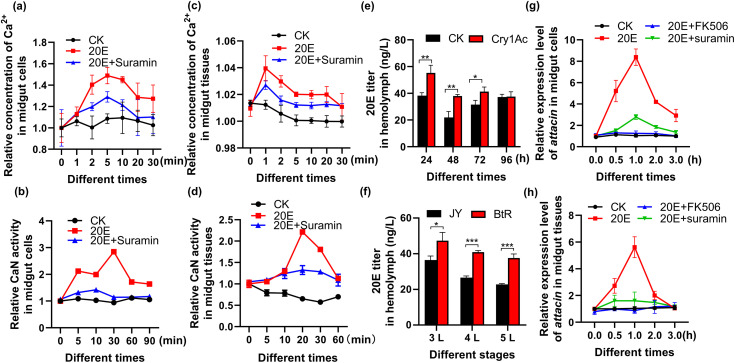
Cry1Ac affected 20E titer, Ca^2+^ concentration and CaN activities. **(a)** The effects of 20E and suramin on intracellular Ca^2+^ concentration in MG cells. Significant differences among the different treatments are indicated in S3 Table. **(b)** The effects of 20E and suramin on CaN activity in MG cells. Significant differences among the different treatments are indicated in S4 Table. **(c)** The effects of 20E and suramin on Ca^2+^ concentration in midgut tissues (n = 5-6). Significant differences among the different treatments are indicated in S5 Table. **(d)** The effects of 20E and suramin on CaN activity in midgut tissues (n = 5 - 6). Significant differences among the different treatments are indicated in S6 Table. **(e)** Variation of 20E titers in the hemolymph of larvae of susceptible strains after feeding Cry1Ac protein (n = 10-15). **(f)** Variation of 20E titer in hemolymph of larvae of resistant and susceptible strains (3rd: n = 25 - 30, 4th: n = 15 -20, 5th: n = 10 - 15). **(g)** Transcription level changes of cellular attacin gene in MG cells in response to 20E, FK506, and suramin (n = 5 - 6). Significant differences among the different treatments are indicated in S7 Table. **(h)** Transcription level changes of the attacin gene in midgut tissues in response to Cry1Ac, FK506, and suramin (n = 5 - 6). CK, control group. Significant differences among the different treatments are indicated in S8 Table. The error bars represent the mean ± SD. Significant differences among the different treatments are indicated by asterisks (**P < 0.01, ***P < 0.001 based on Student’s *t*-tes*t*s, DPS7.05).

To investigate the potential mechanism by which Cry1Ac exposure elevates intracellular Ca^2+^ levels, we investigated a possible link with 20E. This ecdysone hormone is recognized not only for its role in regulating Ca^2+^ concentrations within the midgut but also for activating defense responses against Bt Cry toxins in other insect species [28]. When MG cells or larvae were treated with 20E, a significant increase in both Ca^2+^ concentration (Fig 8a) and CaN enzyme activity (Fig 8b) were observed. Both increases were significantly attenuated when suramin was co-administered (Fig 8c, 8d). Quantification of 20E levels in larvae subjected to Cry1Ac revealed a significant rise compared to controls (Fig 8e; 24 h: *t* = 4.8458, *P* = 0.0084, 48 h: *t* = 6.4697, *P* = 0.0075, 72 h: *t* = 3.6605, *P* = 0.0216, 96 h: *t* = 0.1774, *P* = 0.8678, respectively), with resistant insects exhibiting higher titers than susceptible counterparts (Fig 8f; 3 L: *t* = 3.6590, *P* = 0.0216, 4 L: *t* = 18.1771, *P* *<* 0.001, 5 L: *t* = 11.0680, *P* *<* 0.001, respec*t*ively). Furthermore, exposure of MG cells and tissues to exogenous 20E triggered a transient increase in attacin expression, this effect was significantly reduced upon treatment with either FK506 or suramin (Fig 8g, 8h).

## 3. Discussion

**Fig 9:**
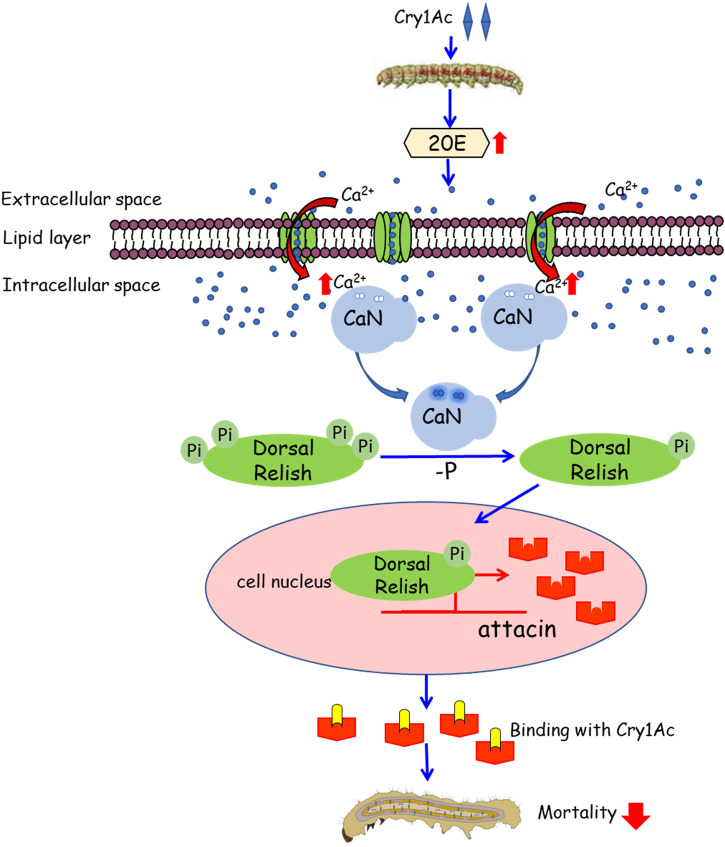
Mechanism of immune action of Cry1Ac in larvae. Cry1Ac treatment leads to an elevation in the level of 20E, subsequently resulting in an increase in intracellular Ca^2+^ concentration. This rise in Ca^2+^ levels activate CaN, which then stimulates attacin expression through transcription factors (relish and dorsal). Ultimately, attacin binds and sequesters Cry1Ac, thereby contributing to resistance against Cry1Ac.

Numerous investigations have highlighted the critical role that intrinsic immune responses in insects play in mitigating the virulence of Bt pesticidal proteins [1–5]. For instance, Cry5B exposure in nematodes induces activation of the p38 and JNK-like mitogen-activated protein kinase (MAPK) signaling pathways, thereby promoting host survival and physiological homeostasis through multiple intracellular innate immune effectors [2–5,8,32]. Additionally, several reports have demonstrated that these immune responses may be transmitted across subsequent generations [6,7]. However, the specific regulatory mechanisms have not been thoroughly elucidated. In this study, we present the first direct evidence of an immune regulatory pathway initiated by Bt exposure in agricultural insects. Specifically, Cry1Ac treatment led to an increase in the 20E titer in cotton bollworm, which subsequently elevated intracellular Ca^2+^ concentration. This rise in Ca^2+^ levels activated CaN, leading to the upregulation of attacin expression of attacin through the transcription factors Relish and Dorsal. Ultimately, attacin interacted with Cry1Ac to attenuate its toxicity against cotton bollworm (Fig 9). Moreover, the resistant strain seems to keep the activated immune regulatory pathway even in the absence of Cry1Ac. This observation parallels findings of reduced Bt receptor gene expression in resistant strains even in the absence of Bt induction [33–35], suggesting that this pathway may form a stable resistance to Cry1Ac. These findings contribute to the robust body of evidence demonstrating that insects can activate immune defense as an adaptive response to the presence of Bt toxins.

In immune regulatory pathway identified in the cotton bollworm, exposure to Cry1Ac results in elevated levels of 20E titers (Fig 7a). Interestingly, larvae that show resistance to Cry1Ac also demonstrate increased 20E titers (Fig 7b), a pattern that corresponds with observations in Cry1Ac-resistant diamondback moth [28]. Further research by Guo et al. (2024) revealed that a reduction in the transcriptional repressor (PxDfd) increases the expression of a midgut microRNA (miR-8545) [36], which in turn suppresses the expression of a newly identified ecdysteroid-degrading glucose dehydrogenase (PxGLD). Downregulation of PxGLD reduces 20E degradation, thereby increasing 20E levels. Although the exact mechanism by which 20E levels in cotton bollworms rise in response to Cry1Ac induction remains unknown, these finding suggests a non-random association between Bt protein exposure and subsequent 20E-mediated signaling cascades in Lepidoptera. It should be noted, however, the observed increase in Ca^2+^ may not be solely due to the elevated levels of 20E. Suramin has been shown to effectively suppressed the activation of heterotrimeric G proteins in various GPCRs, including ErGPCR, which serves as the 20E receptor on the membrane. When midgut tissues were exposed to 20E or Cry1Ac, there was an increase in Ca^2+^ concentration, CaN activities and attacin expression (Figs 7 and 8). These data support the involvement of a 20E/Ca^2+^/CaN/attacin signaling axis in mediating Cry1Ac toxicity in cotton bollworm. However, suramin could not fully abolish the elevated Ca^2+^, CaN activity, and attacin expression (Figs 7 and 8), indicating that alternative pathways may also contribute to Ca^2+^ influx. Further evidence from isolated midgut cell lines (Figs 7 and 1e) demonstrated that Cry1Ac induced increased Ca^2+^ concentration, elevated CaN activity, and attacin overexpression in the isolated MG cell line, suggesting alternative pathways might facilitate Ca² ⁺ elevation. After all, 20E synthesis has been reported to mainly occur in larval prothoracic gland or embryonic cells [37], while whether MG produces 20E remains need to be explored, but other calcium sources warrant consideration. Previous studies have shown that the Endoplasmic reticulum (ER) acts as a crucial reservoir for Ca^2+^. Moreover, Bt δ-endotoxins have been shown to affect the ER, resulting in ER cisternae swelling and the formation of numerous small discrete vesicles or large vacuoles within the rough ER [38–40]. Consequently, it is possible that Cry1Ac prompts the release of Ca²⁺ from the ER into the cytoplasm. The influx of Ca^2+^ from alternative pathways may potentially modulate CaN enzymatic activities and facilitate the upregulation of attacin expression. Although the increase in Ca² ⁺ concentration and the upregulation of attacin expression induced by Cry1Ac may involve additional signaling pathways, the involvement of 20E/Ca² ⁺ /CaN/attacin pathway in cotton bollworm defense against Cry1Ac is evident. Importantly, Cry1Ac was found to activate these signaling cascades, consistent with previous findings, CaN inhibition suppresses core immunity in cotton bollworm and improves Bt insecticidal efficacy [9–13,30,41,42].

In model organisms, CaN facilitates innate immune responses by dephosphorylating and activating transcription factors, there by initiating the expression of relevant AMP genes through the Toll and IMD pathways [12,43,44]. Moreover, previous studies have indicated that CaN also regulates transcription factors such as NFAT, Relish, Dors, and Dif [13,44,45]. Notably, the specific immune pathways and transcription factors influenced by CaN appear to vary depending on the pathogen encountered. Our previous studies have revealed that CaN binds to Relish and regulates the expression of AMPs, including attacin [13]. In the current study, the Bt-derived secreted protein Cry1Ac was utilized, revealing that Relish induces attacin expression (Fig 4a). Moreover, overexpression of Relish elevated attacin levels and concurrently mitigated Cry1Ac-induced cytotoxicity (Fig 4b). These observations are consistent with the findings from RNAi-mediated knockdown of Relish expression in *L. migratoria*, where Relish knockdown significantly reduced attacin production [46]. Furthermore, CaN also binds and dephosphorylates Dorsal. Inhibition of CaN activity prevented this dephosphorylation (Fig 5g). Dorsal, in turn, targets the attacin promoter, and its overexpression enhances attacin expression while diminishing Cry1Ac cytotoxic effects (Fig 9). Moreover, attacin could be induced both gram-positive and gram-negative bacteria exhibiting antibacterial activity against these pathogens [47]. The findings from this study demonstrate that CaN regulates both Relish and Dorsal transcription factors, thereby controlling attacin expression in *H. armigera*. Nonetheless, attacin regulation appears to be complex, involving additional signaling pathways. Indeed, Bt has been shown to activate other immune pathways such as the JAK/STAT, MAPK signaling pathway [2–5,8,28,32,48,49], which can also influence the expression of AMPs[1]. Future research should investigate other pathways contributing to attacin upregulation in response to Cry1Ac and assess whether other AMPs similarly alleviate Cry1Ac-associated toxicity.

It has been observed that Bt toxins can induce the expression of attacin in *L. migratoria* [47]. However, research focusing specifically on the role of attacin in mediating the effects of Bt pesticidal proteins remains scarce. Several potential mechanisms may underlie this interaction. Firstly, previous investigations have demonstrated that Bt infection and Cry toxins can significantly reduce gut microbial diversity and abundance in *Galleria mellonella* [50],*Plutella xylostella* [15], and *L. migratoria* [47]. In our experiment feeding larvae with attacin protein, possibly experienced alterations in their gut microbiota as a defensive response against Cry1Ac. However, we speculated that such changes in the microbial community did not significantly reduce the toxicity of Cry1Ac, given that high doses of attacin protein did not significantly influence larval weight or mortality rates (Fig 3f, 3g). Secondly, the reduction in Cry1Ac toxicity induced by attacin might primarily occur through direct binding between attacin and Cry1Ac, which could lower the concentration of Cry1Ac in the midgut. This decrease would reduce Cry1Ac’s receptor binding, thereby diminishing its toxic effects. Supporting this possibility, similar studies have reported that glycolipid bind to Cry1Ac to make it inactivation by a coagulation reaction [51,52]. Likewise, in *Caenorhabditis elegans*, increases the expression of galectin LEC-8 to inhibit Cry5B-glycolipids (glycolipids are receptors of Cry5B in these two APNs) binding interaction to remove pores formed by Cry5B [53]. Alternatively, attacin binding may not obstruct Cry1Ac from engaging receptors but could generate steric hindrance that modifies Cry1Ac’s conformation, impairing its ability to form pores, a key aspect of the Bt mechanism of action [54]. Irrespective of the exact mechanism involved, this study suggests that suppressing CaN-regulated immune responses could serve as an effective target for enhancing the efficacy of Bt insecticides and managing resistance.

## 4. Materials and methods

### 4.1. Insect, cell culture and Cry1Ac protein

Two laboratory strains of *H. armigera* were utilized in this study. The susceptible strain purchased from Henan Jiyuan Baiyun Industry Co. Ltd. (named JY) was raised in the laboratory on an artificial diet without insecticides. The BtR strain was selected through exposure to Cry1Ac protoxin and exhibited resistance levels exceeding 3000-fold [55].

The *H. zea* midgut cell line (MG cells), generously provided by Dr. Cynthia L. Goodman (BCIRL, USDA, ARS), was maintained in a constant temperature incubator at 28°C using EX-CELL 420 insect serum-free medium (SAFC Bioscience, Lenexa, KS) as previously described [9,30,19]. The *Spodoptera frugiperda* ovary cell line (Sf9 cells) was cultured at 28°C in Sf-900 IISFM culture medium, supplemented with 10% fetal bovine serum (FBS) and 0.5% Penicillin-Streptomycin from the Gibco Company (Gibco, St, USA). Sf9 cell line is a well-established model for high-efficiency heterologous protein expression using the pIEx vector system. The HEK293T cell line was cultured in Duchenne’s modified eagle’s medium and placed in a constant temperature incubator at 37°C containing 5% CO_2_.

The Cry1Ac protoxin and activated Cry1Ac toxins were generously supplied by Insect-Resistant Biotechnology Laboratory, Institute of Plant Protection, Chinese Academy of Agricultural Sciences. The activated Cry1Ac proteins were then reconstituted in EX-CELL 420 insect serum-free medium following the protocols outlined in our previous publications for conducting cytotoxicity assays [19].

### 4.2. Measurement of CaN enzymatic activities and transcription levels of the attacin gene

Samples from different stages of Cry1Ac-susceptible and resistant strains. Larvae of Cry1Ac-susceptible and resistant strains were reared on a non-Cry1Ac-containing artificial diet. Midguts from 3rd (10–15), 4th (6–10), and 5th (6–10) instar larvae were dissected, and the gut contents carefully removed on ice. The cleaned midgut tissue was immediately flash-frozen in liquid nitrogen. Each sample included three replicates, consisting of larvae from the same stages and different strains.

Larvae samples treated by Cry1Ac. For the JY strain, a concentration of 25 μg/mL Cry1Ac was used as it resulted in approximately 30% mortality for fifth instar larvae [30]. Prior to exposure to artificial diets containing Cry1Ac, fifth instar larvae of the same size underwent a 12-h starvation period. Subsequently, midguts (4–5 larvae) corresponding to each treatment were collected at the afore mentioned time points. Each treatment was replicated three times.

MG cells and midgut tissues treated by Cry1Ac. MG cells were seeded at a density of approximately 4.5 × 10^5^ cells per well in 24-well plates and allowed to fully attach for over 2 hours in a 28 °C incubator. Each well was treated with either Cry1Ac (30 μg/mL) or 20E (5 μM) at different time points. Fifth instar larvae with clean midguts of the same size were obtained, and their midguts (n = 5–6) were collected and incubated with Graces’ medium at 28 °C for 2 h. Each well was treated with Cry1Ac at different time points, using Na_2_CO_3_ as the control. Each well was treatment with Cry1Ac (30 μg/mL) or 20E (5 μM) at different times. Three biological replicates were performed, with each replicate containing at least three wells. The samples were incubated with suramin (20 μM) and FK506 (10 μM) (for 1 h) before being subjected to CaN enzymatic activity measurement using the CaN enzymatic activity kit (Nanjing Jiancheng Biotechnology Research Institute Co., Ltd.). Three biological replicates were conducted.

### 4.3. Real-time quantitative PCR

Total RNA was isolated according to the instructions provided by the RNA-easy isolation reagent Kit (Vazyme Biotech Co., Ltd.). Subsequently, first-strand cDNA synthesis was performed using the HiScript II QRT SuperMix for qPCR (+gDNA wiper) Kit (Vazyme Biotech Co., Ltd.). Primer sequences were designed using Primer Version 5.00 and are listed in S9 Table. Quantitative real-time PCR (qPCR) was conducted to determine the mRNA levels of candidate genes using the ChamQ Universal SYBR qPCR Master Mix Kit (Vazyme Biotech Co., Ltd.). The reaction system and qPCR program followed protocol in Yao et al. 2023 [56]. Two reference genes (EF-1α: GenBank U20129.1 and actin: GenBank HM629442.1) from *H. armigera* were selected as internal controls for normalizing the expression of candidate genes. Each experiment consisted of three biological replicates, with each biological replicate including three technical replicates. Statistical analysis was performed using either Student’s *t*-test or Turkey test (DPS7.05) to compare significant differences.

### 4.4. cDNA cloning and Construction of plasmids

The midguts of 5th instar larvae were used for total RNA extraction using an RNAiso plus kit (Takara Biomedical Technology Co., Ltd.) following the manufacturer’s instructions. The integrity of the extracted RNA was assessed by agarose gel electrophoresis, and its quantity was determined using a NanoDrop 1000 spectrophotometer (Thermo Fisher Scientific Inc.). For gene cloning, cDNA synthesis was performed using the HiScript III RT SuperMix for qPCR (+gDNA wiper) (Vazyme Biotech Co., Ltd.). The synthesized cDNA was immediately stored at -20°C until further use. Nucleotide sequences of *H. armigera* genes were obtained from GenBank database [NCBI accession numbers: KR185962.1 (CaN); JN315687.1 (Dorsal); JN315690.1 (Relish); AY948540.1 (attacin)]. Specific primers for gene cloning were designed using Primer Premier 5.0 and PCR amplicons from *H. armigera* were cloned into ZT4 vector (Beijing Zoman Biotechnology Co., Ltd), followed by transformation into *Escherichia coli* DH5α competent cells (Tsingke Biotechnology Co., Ltd) for sequencing. The full-length open reading frame (ORF) of CaN and Dorsal/Relish or attacin were cloned into the pET-30a and pIEx-GFP/RFP-His vectors, respectively. ORFs of CaN and Dorsal/Relish or attacin without a translation stop codon mutation (TAA to TAC) was generated by PCR using CaN and Dorsal or attacin-ZT4 as templates, with the primers listed in S9 Table for plasmid construction. Additionally, the pIEx-GFP/RFP-His vector was digested with Bgl II and Sac I at 37°C for 2 h. GFP/RFP-Dorsal/Relish or attacin-F/R primers containing Bgl II and Sac I sites (S9 Table) were designed to amplify the corresponding ORFs of Dorsal or attacin encoding amino acid residues 564 or 207 from bases 1695 bp or 624 bp, respectively. The same method was applied for cloning the CaN ORF into the pET-30a vector. Subsequently, PCR products were purified using a DNA gel extraction kit (Vazyme Biotech Co., Ltd.) and subcloned into the pIEx-GFP/RFP-His vector as well as into the His-tag expression vector separately. Ligation reactions between vectors and target genes were carried out at 37 °C for 30 min using ClonExpress II One Step Cloning Kit (Vazyme Biotech Co., Ltd.), followed by transformation into *E. coli* DH5-ɑ cells (Tsingke Biotechnology Co., Ltd.). Finally, sequencing confirmed the presence of recombinant plasmids.

### 4.5. Protein expression, purification and obtain antibodies

The pET-30a plasmid containing the CaN/attacin gene was introduced into Rosetta (DE3) cells. Following ampicillin screening, single colonies were selected and cultured in LB medium supplemented with ampicillin at 37 °C and shaken at 220 r/min until reaching an OD600 of 0.4–0.6. Some samples were collected for SDS-PAGE analysis. Subsequently, bacterial cells were induced with 0.2 mM isopropyl β-D-1-thiogalactopyranoside (IPTG) at 16 °C for 16 h to initiate fusion protein expression, and samples were collected for SDS-PAGE analysis. The expressed protein was confirmed using a His-tag antibody specific to the desired protein target. After harvesting the bacteria, they were resuspended in 30 mL PBS with the addition of 30 μL β-mercaptoethanol and 300 μL PMSF before being sonicated on ice for 15 min (amplitude: 20%, on/off cycle: 5 s). The resulting supernatant containing soluble albumin proteins was transferred to a new tube while performing three washes of inclusion bodies as follows: Firstly, inclusion body proteins were pelleted by centrifugation at 12000 rpm at 4°C for 10 min. The supernatant was discarded and the inclusion body proteins were resuspended in lysis buffer (20mM PBS,150mM NaCl, pH7.4) containing TritonX-100(0.5-1%) followed by another centrifugation step under similar conditions. The inclusion body proteins were then resuspended in lysis buffer (20 mM PBS, 150 mM NaCl, pH 7.4), sonicated on ice for 15 minutes, and subjected to centrifugation again under the same conditions, with the supernatant retained after each wash step. After washing, the inclusion bodies were lysed using 10 mL lysis buffer (20 mM PBS, 150 mM NaCl, 8 M Urea, pH7.4) containing β-ME (0.1%), followed by centrifugation (stepwise parameters same as above), and confirmed via SDS-PAGE. The candidate proteins were subjected to gradient dilution using Urea (8 M, 4 M, 2 M, 1M and 0 M). Subsequently, CaN protein was dissolved in buffer (pH7.4) containing PBS, 1M Urea and 10% Glycerol, while attacin protein was dissolved in buffer (pH7.4) containing PBS. Finally, the CaN/attacin proteins were concentrated using Ultra-15 Centrifugal Filters (Merck Millipore Ltd. Tullagreen, Carrigtwohill, Co Cork IRL), and their concentrations determined using a BCA Protein Assay Kit (Nanjing Jiancheng Biotechnology Research Institute Co., Ltd.). Antibodies against the CaN protein was obtained by injecting protein into New Zealand rabbits.

### 4.6. Western blot

Total protein was extracted using RIPA buffer (Solarbio), followed by separation on a 10% SDS-PAGE gel (Shaanxi Zhonghui Hecai Biomedical Technology Co., Ltd). Subsequent detection was performed with a CaN-specific polyclonal antibody (diluted 1:150). Image visualization was achieved using an ECL buffer (Abbkine Scientific Co., Ltd).

### 4.7. Protein-protein docking

The structures of proteins were download from Uniprot. The structures of attacin (A0A2W1BU65)/Dorsal (G3LF42) and CaN (A0A0U2JFS6) were predicted using Alphafold. The structure of Cry1Ac (P05068) had been obtained by X-ray crystallography. To ensure the accuracy of the docking results, the protein was prepared using AutoDockTools-1.5.7 [57], with manual removal of water molecules and the addition of polar hydrogen atoms. Protein-protein docking was performed using the Docking Web Server (GRAMM) [58,59]. The resulting protein-protein complex was further optimized by removing water molecules and adding polar hydrogen atoms using AutoDockTools-1.5.7. Finally, PyMOL was used to predict protein-protein interactions and generate a fig illustrating these interactions. The attacin/Dorsal complex is depicted as a slate-colored cartoon model, while the Cry1Ac/CaN one is shown as a cyan-colored cartoon model, with their respective binding sites represented as stick structures in corresponding colors. When focusing on the binding region, only the relevant protein involved in each binding site is displayed.

### 4.8. Ligand blot

Twenty microliters of attacin protein (0.1 μg/mL) were resolved by 10% SDS-PAGE (CFAS any KD PAGE). An equivalent amount of BSA was employed as a control. The proteins were then transferred onto a PVDF membrane (polyvinylidene fluoride, Merck Millipore Ltd). The transferred proteins on the PVDF membrane were blocked overnight using a blocking buffer solution containing 5% BSA in PBST. Following this, Cry1Ac protoxin or activated Cry1Ac at a final concentration of 2 μg/mL was added and incubated for 2 hours. After washing five times with PBST for 5 min each time, the membrane was treated with a dilution of Cry1A antibody at a ratio of 1:10000. Further washing steps with PBST were performed before incubating the membrane with sheep anti-rabbit IgG antibody for 1 h. After additional washes, the membrane was developed using SuperKine West Femto Maximum Sensitivity Substrate (Abbkine Scientific Co., Ltd) and exposed on the Tanon-4600 photographic system.

### 4.9. Homologous competition

The production of biotinylated Cry1Ac was conducted following the established protocol [60]. Ligand blotting method, as described above, was employed with HRP−streptavidin antibody (Abbkine Scientific Co., Ltd) capable of recognizing biotinylated Cry1Ac [56,61].The ligand blot method described above was used for both saturation and competition experiments with some modifications. In the saturation experiment, 2 μg of attacin protein was separated on an SDS-PAGE gel and then transferred to a PVDF membrane. The PVDF membranes were cut into 4 pieces, each with one maker lane and one attacin lane 4 pieces of membranes were incubated with 5% BSA overnight, and then 1, 2, 5, 10, or 15 μg of biotinylated activated Cry1Ac was added, and the membranes were incubated for 4 h. The intensities of attacin and biotinylated activated Cry1Ac bands on the 4 blots were quantified by Image J software. The saturation experiment was repeated three times and showed that 2 μg of purified attacin saturated with 5 μg of biotinylated activated Cry1Ac. The homologue competition experiments were performed in a similar way to the saturation experiments. After membrane transfer, the membranes were co-incubated for 4 h with 5 μg of biotinylated activated Cry1Ac and 25 μg (5-fold) or 50 μg (10-fold) of homologous competitor (unlabeled activated Cry1Ac), respectively.

### 4.10. Cells transfection

Sf9/MG cells were seeded into 24-well plates at a density of approximately 4.5 × 10^5^ cells per well and subsequently incubated in a 28 °C incubator for full attachment (>2 h). Each well was then transfected with 1 μg of plasmid using Cellfectin (Promega Biotech Co., Ltd) and incubated for 5 h. Subsequently, replace the culture medium with serum containing culture medium for cultivation. Take photos and record the expression of fluorescent proteins inside the cells. Forty-eight hours post-transfection, the treated cells were subjected to a cytotoxicity test with Cry1Ac toxin (200 μg/mL for Sf9 cells and 30 μg/mL for MG cells). pIEx- RFP-His and pIEx- attacin- RFP-His cells were transfected with Sf9 cells, and pIEx-GFP/RFP-His and pIEx-Relish/Dorsal -GFP/RFP-His cells were transfected with MG cells. Details of the cell bioassay is referred to in our previous publications [9,30,19]. Finally, cell mortality was calculated using an inverted microscope (LEICA DMi8). Each treatment was repeated 3–6 times, with three randomly selected views per well. The remaining treated cells of each independent transfection were collected for protein extraction to confirm successful transfection as described in our previous description [9,30,19].

### 4.11. Cell bioassay

pIEx-GFP/RFP-His and pIEx-Relish/Dorsal/attacin-GFP/RFP-His cells were observed and photographed 24 hours post-transfection. Forty-eight hours after transfection, the treated cells were subjected to a cytotoxicity test with Cry1Ac toxin. Detailed information on the cell bioassay is referred to in our previous publications [9,30,19]. Cell mortality was then measured using an inverted microscope (LEICA DMi8). Each treatment was repeated 3–6 times with three randomly selected views per well. Remaining treated cells from each independent transfection were collected for protein extraction to confirm the successful transfection, as described in our previous work [9,30,19].

### 4.12. Larval bioassays

For verifying the effect of excess attacin on Cry1Ac toxicity in a susceptible strain, diet overlay bioassays was used to determine the susceptible of newly hatched larvae to attacin at various concentrations (PBS buffer used to dissolve attacin: 0.025 (1×), 0.125 (5×) and 0.375 (15×) μg/cm², as well as mixtures of Cry1Ac and attacin (PBS alone, Na_2_CO_3_ in PBS, 0.025 μg/cm² Cry1Ac in PBS, 0.375 (15×) μg/cm² attacin in Na_2_CO_3_, 0.025 μg/cm² Cry1Ac with 0.375 (15×) μg/cm² attacin). The concentration in this experiment was set by fixing the Cry1Ac toxin and adjusting the proportion of attacin, which was used to test the maximum protective potential of attacin against Cry1Ac toxicity under conditions of attacin excess, as well as to assess the potential adverse effects of exogenously administered attacin on larval physiology. This method and the chosen ratio were based on previously reported literature [62–64]. Newly hatched larvae of the same size (less than 6 h old) were selected and inoculated into the prepared 24 well plates. 3–5 biological replicates were employed, with each replicate consisting of 24 larvae. The weights of the larvae that treated by attacin were calculated, and the mortality of each treatment was recorded at the 7th days.

### 4.13. Dual-luciferase reporter assay

The constructed plasmids were divided into four treatment groups: psicheck+pcDNA3.1C, psicheck+pcDNA3.1-Dorsal, psicheck-attacin +pcDNA3.1-Dorsal and psicheck-attacin+ pcDNA3.1-Dorsal. Theses plasmids were transfected into HEK 293T cells, and the luciferase activity in the samples was detected using a dual luciferase reporter gene assay kit (Yeasen, 11405ES60) after 48 hours of culture.

### 4.14. Phosphorylation level analyses of Dorsal

The pIEx-Dorsal-GFP-His plasmid (5 μg) was transfected into MG cells (approximately 3.6 × 10^6^ cells per well) using FuGENE HD Transfection Reagent (Promega, E2311). Forty-eight hours post-transfection, as previously described (Southern & Berg, 1982), MG cells were incubated with Cry1Ac (30 μg/mL) for various durations (0, 10 and 30 minutes). Similarly, MG cells were pre-treated with the CaN inhibitor FK506 (10 μM) for 1 hour, followed by incubation with Cry1Ac (30 μg/mL) for different time intervals (0, 10 and 30 minutes). Protein extraction was performed using RIPA lysis buffer, and the protein concentration was determined using the BCA Protein Assay Kit (Nanjing Jiancheng Biotechnology Research Institute Co., Ltd.). A portion of the lysate (60 μL) was used as input. The residual lysate was then incubated with phosphoserine antibody (Abcam, ab9332) at 4°C for 8 hours with rotation according to the manufacturer’s instructions. Subsequently, Pierce Protein A/G Magnetic beads from ThermoFisher were mixed with the samples for a duration of two hours. Finally, western blot analysis was conducted on the immunoprecipitation solution using His antibody from Abbikine.

### 4.15. Coimmunoprecipitation

The ORF sequence of CaN was ligated into the pIEx-RFP-His vector to construct the pIEx-CaN-RFP-His plasmid. MG cells were cotransfected with pIEx-Dorsal-GFP-His and pIEx-CaN-RFP-His plasmids (one Co-IP experiment with 7.2 × 10^6^ cells and 4 μg of each plasmid). After 48 hours, the cells were collected and lysed on ice using 600 μL Radio Immunoprecipitation Assay Lysis buffer containing a protease inhibitor. The protein concentration was determined using a BCA Protein Assay Kit (Nanjing Jiancheng Biotechnology Research Institute Co., Ltd.). A total of 60 μL input was taken, and the remaining solution was divided into two equal parts. One part was incubated with anti-GFP antibody (Abbkine Scientific Co., Ltd, ABM40124) at 4 °C for 8 h, followed by incubation with Pierce Protein A/G Magnetic beads (Thermo Fisher Scientific Inc., 88802) for 2 h to purify the target GFP-tagged proteins. Subsequently, the protein samples were transferred onto a polyvinylidene fluoride membrane and detected using RFP (Bioworld, MB2015) or GFP antibodies through Western blotting analysis. Additionally, protein samples were analyzed by SDS-PAGE followed by Coomassie brilliant blue staining.

### 4.16. The measurement of 20-hydroxyecdysone contents

Samples of different stages of Cry1Ac-susceptible and resistance larvae. Larvae of JY and BtR strains were reared on a non-Cry1Ac artificial diet. Hemolymph samples from the 3rd (25–30), 4th (15–20), and 5th (10–15) instars were dissected, followed by removal of gut contents on ice. Samples after Cry1Ac treatment on larvae were treated as described above. Subsequently, hemolymph tissues from 10-15 larvae for each treatment were harvested at different time points. Each treatment was replicated three times. The measurement of 20E contents was performed using the 20E contents kit (Shanghai Enzyme-linked Biotechnology Co., Ltd.) according to the manufacturer’s instructions. Three biological replicates were utilized.

### 4.17. The measurement of Ca^2+^ concentration

The acquisition of MG cell samples followed the same procedure as for CaN enzymatic activity samples. The Ca^2+^ concentration was measured using the Fluo-3, AM Calcium Ion Detection Kit (CA1180, Solarbio) following the manufacturer’s instructions [65]. Three biological replicates were employed, with each replicate containing at least five wells. Fifth instar larvae of the same size were obtained as previously described and subjected to Ca^2+^ concentration by the Calcium Ion Assay Kit (Beyotime Biotechnology) following the manufacturer’s instructions. Three biological replicates were performed. The inhibitors used for sample incubation were suramin (20 μM) and FK506 (10 μM).

### 4.18. Statistical analysis

The statistical analyses were performed using GraphPad Prism 9, and the data were presented as mean ± standard deviation (SD). Student’s *t*-test was employed for comparing two groups, while One-way analysis of variance (ANOVA) was used when comparing more than two groups. To compare significant differences in relative binding ability, relative expression levels, larval weight, and mortality among different treatments, the Tukey test (DPS7.05) was applied. Cell mortality was calculated according to references [13,66]. Statistically significant differences between groups were indicated within the figures with *, **, and *** denoting *P <* 0.05, < 0.01, and < 0.001 respectively. The different capital letters on the error bars indicate significant differences analyzed using ANOVA and a Tukey test at the level of *P <* 0.01.

## Supporting information

S1 Table Turkey test to compare significant differences by DPS7.05 for Fig 7c. (DOCX)

S2 Table Turkey test to compare significant differences by DPS7.05 for Fig 7d. (DOCX)

S3 Table Turkey test to compare significant differences by DPS7.05 for Fig 8a. (DOCX)

S4 Table Turkey test to compare significant differences by DPS7.05 for Fig 8b. (DOCX)

S5 Table Turkey test to compare significant differences by DPS7.05 for Fig 8c. (DOCX)

S6 Table Turkey test to compare significant differences by DPS7.05 for Fig 8d. (DOCX)

S7 Table Turkey test to compare significant differences by DPS7.05 for Fig 8g. (DOCX)

S8 Table Turkey test to compare significant differences by DPS7.05 for Fig 8h. (DOCX)

S9 Table List of primers in this study. (DOCX)

S1 Fig Expression of CaN protein and preparation of antibodies. (a) SDS-PAGE analysis of CaN protein expression induced by IPTG. (b) Western blot analysis to verify the expression of CaN protein.
(DOCX)

S1 Raw Images S2l Fig. Saturation binding assays between attacin and activated Cry1Ac. S2m Fig. Homologous competition between labeled Cry1Ac and unlabeled Cry1Ac to attacin. S5c Fig. Co-IP experiment between Dorsal and CaN. S5d Fig. Co-IP experiment indicating how much Dorsal protein has been pulled down by an anti-phosphoserine antibody following Cry1Ac treatment. S5e Fig. As above 5d but in the presence of FK506.
(PDF)
