# Supplementary material for: Calcineurin-dependent 20E signaling pathway enhances attacin expression to prime the immunity of Helicoverpa armigera against Bacillus thuringiensis Cry1Ac toxin
Source: PLoS Pathog. 2026 Aug 7;22(8):e1014503. doi: 10.1371/journal.ppat.1014503 (PMC13472496; doi:10.1371/journal.ppat.1014503)
Supplement: Supplementary file 1 [file ppat.1014503.s011.pdf]

S1_raw_images

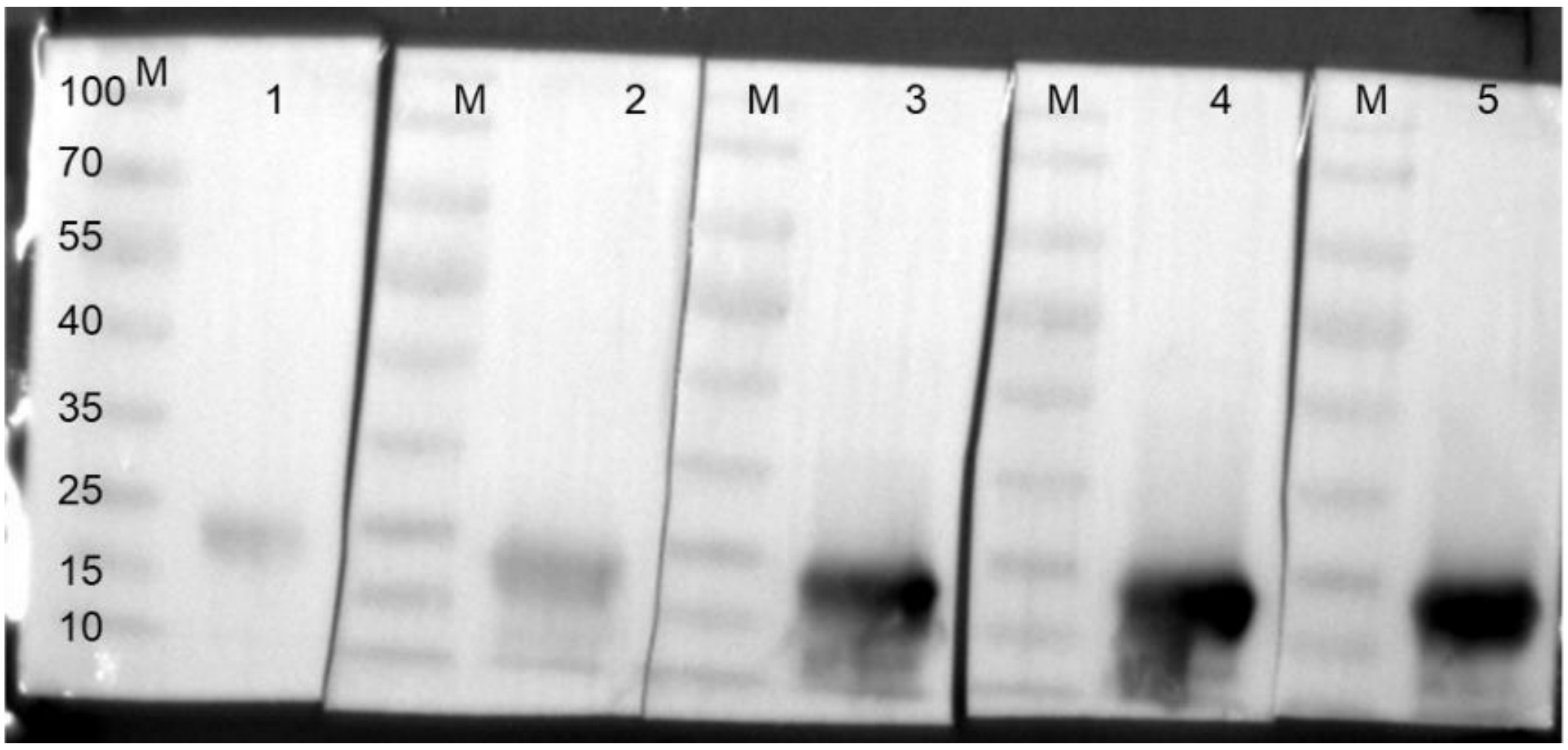

Fig S2l. Saturation binding assays between attacin and activated Cry1Ac. M: protein marker; 1/2/3/4/5: purified attacin-His.

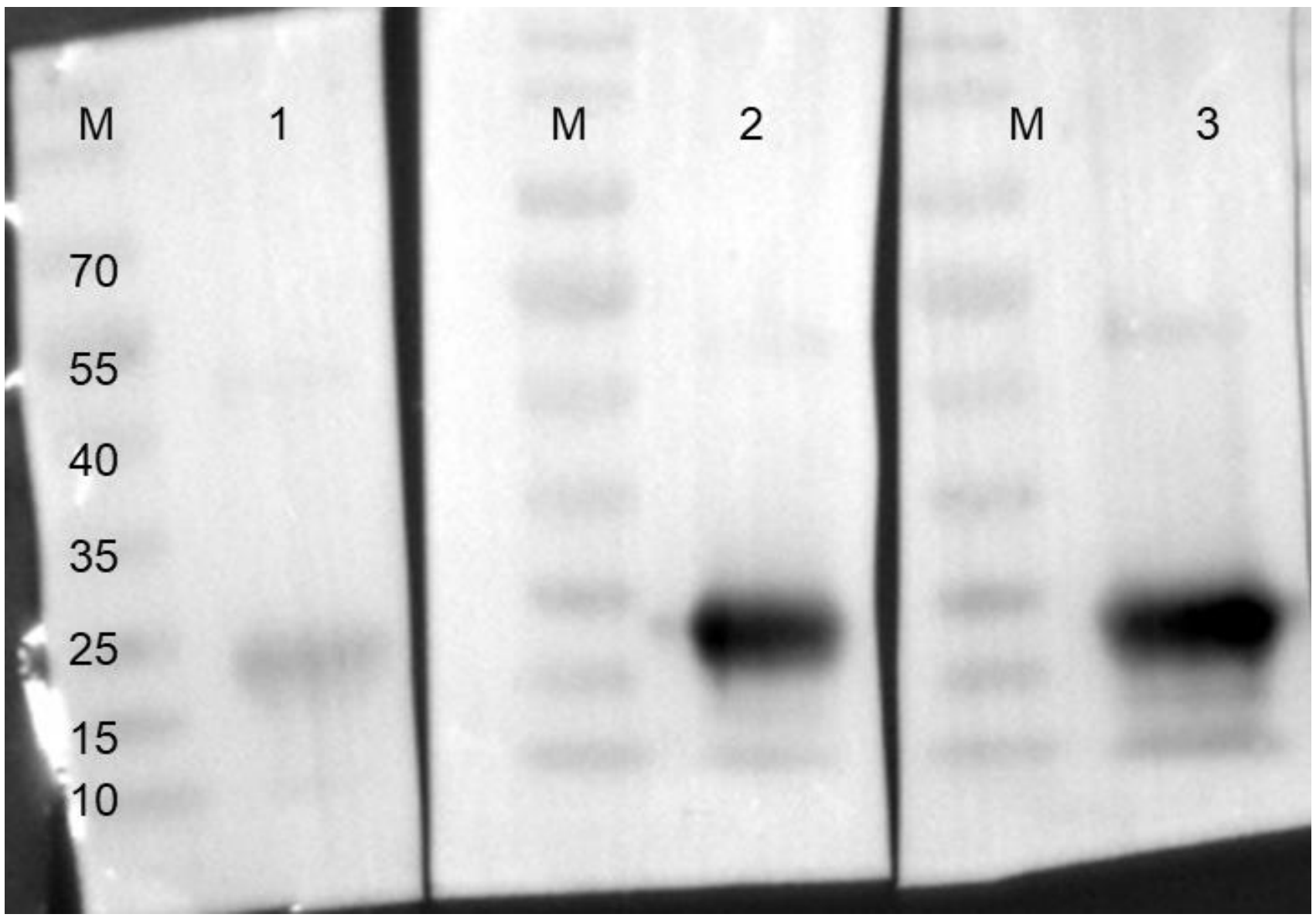

Fig S2m. Homologous competition between labeled Cry1Ac and unlabeled Cry1Ac to attacin. M: protein marker; 1/2/3/: purified attacin-His.

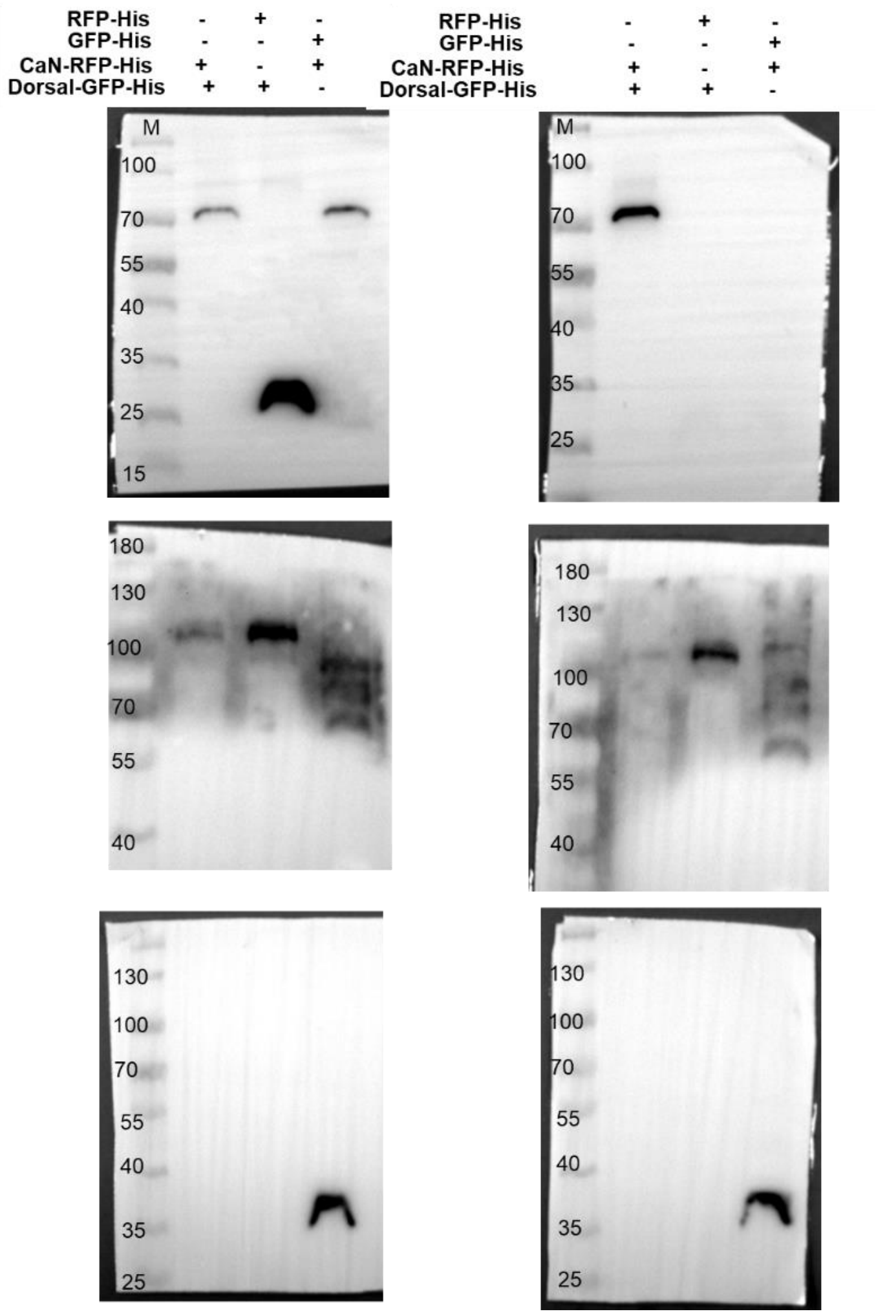

Fig S5c. Co-IP experiment between Dorsal and CaN. The input blots indicate whether the transfected plasmid has successfully expressed the protein in the cells. IP indicates which GFP-containing proteins have been pulled down by the anti-GFP antibody. Co-IP indicates which RFP-containing proteins have been pulled down alongside the GFP ones.

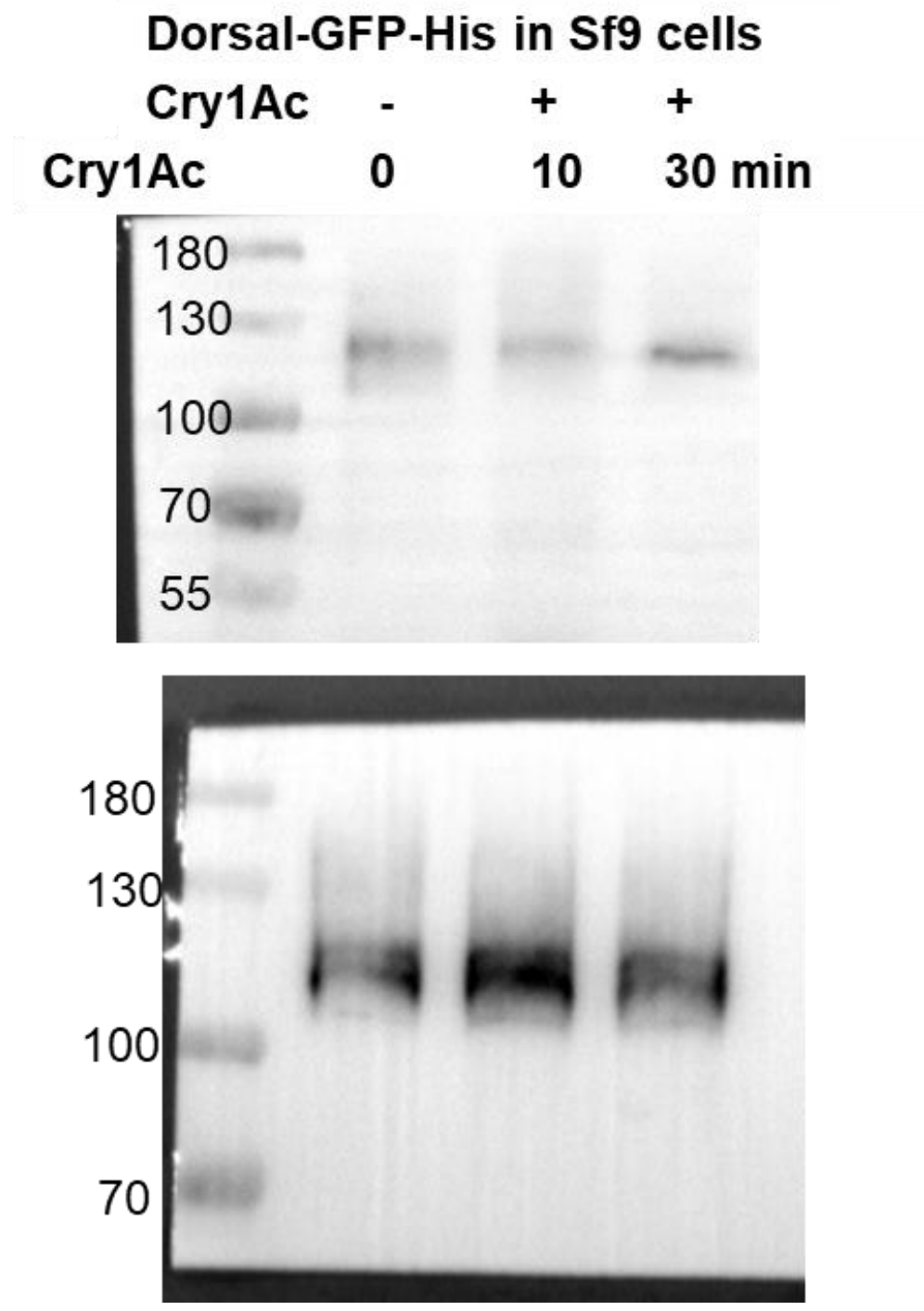

Fig S5d. Co-IP experiment indicating how much Dorsal protein has been pulled down by an anti-phosphoserine antibody following Cry1Ac treatment.

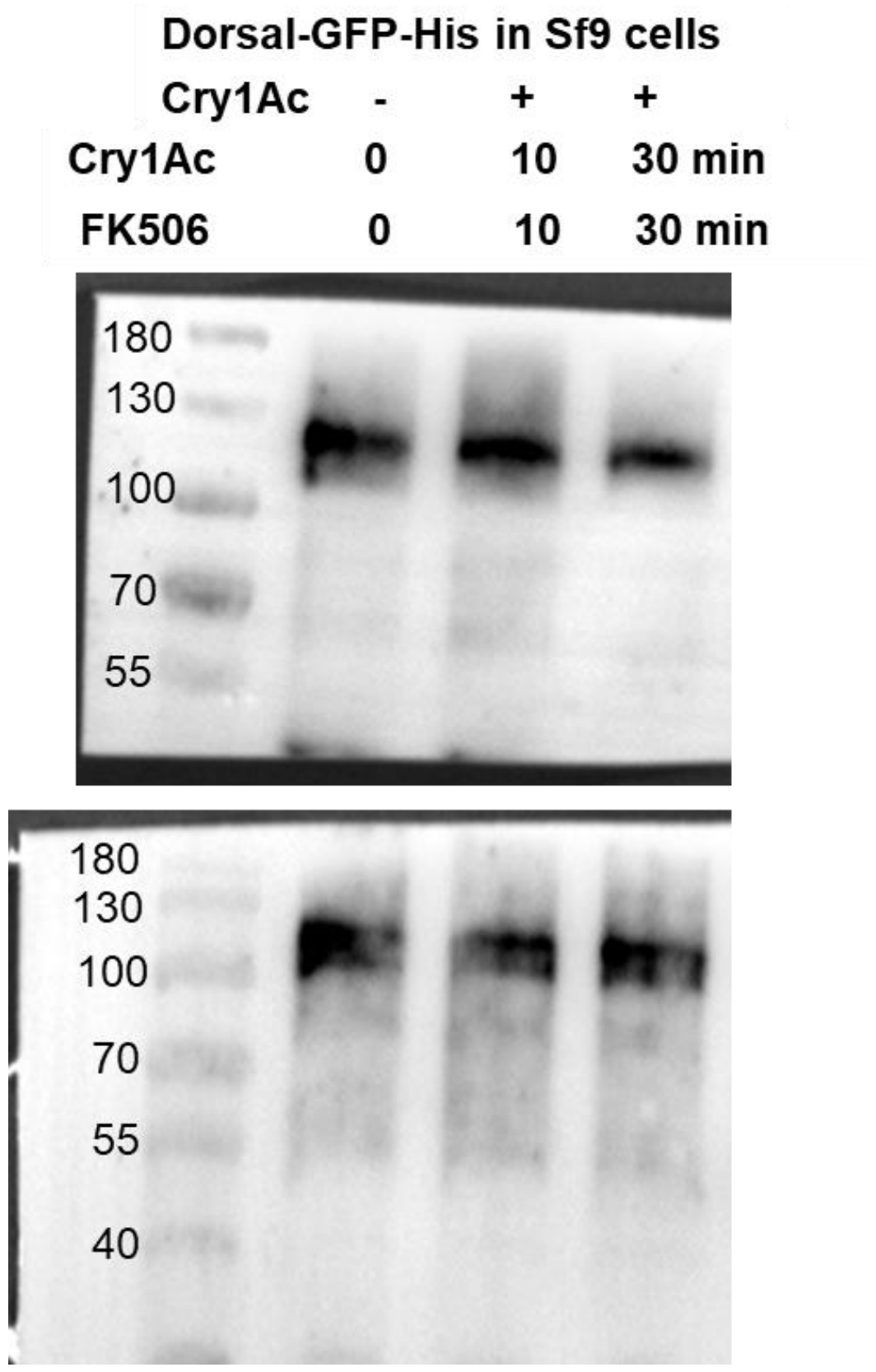

Fig S5e. As above 5d but in the presence of FK506.
