# Supplementary material for: Calcineurin-dependent 20E signaling pathway enhances attacin expression to prime the immunity of Helicoverpa armigera against Bacillus thuringiensis Cry1Ac toxin
Source: PLoS Pathog. 2026 Aug 7;22(8):e1014503. doi: 10.1371/journal.ppat.1014503 (PMC13472496; doi:10.1371/journal.ppat.1014503)
Supplement: Supplementary file 2 [file ppat.1014503.s001.docx]

Table S1 Turkey test to compare significant differences by DPS7.05 for Figure 7c

| Sample | 0 | 0.5 | 1 | 2 | 3 |
| --- | --- | --- | --- | --- | --- |
| CK | A | B | B | B | B |
| Cry1Ac | A | A | A | A | A |
| Cry1Ac+FK506 | A | B | B | B | B |
| Cry1Ac+suramin | A | AB | B | B | B |
| *F* | 0.285 | 10.911 | 157.745 | 103.684 | 35.752 |
| *P* | 0.8353 | 0.0034 | 0.0001 | 0.0001 | 0.0001 |

Note：Different letters indicate significant differences by Turkey test.
