# Supplementary material for: Calcineurin-dependent 20E signaling pathway enhances attacin expression to prime the immunity of Helicoverpa armigera against Bacillus thuringiensis Cry1Ac toxin
Source: PLoS Pathog. 2026 Aug 7;22(8):e1014503. doi: 10.1371/journal.ppat.1014503 (PMC13472496; doi:10.1371/journal.ppat.1014503)
Supplement: Supplementary file 4 [file ppat.1014503.s003.docx]

Table S3 Turkey test to compare significant differences by DPS7.05 for Figure 8a

| Sample | 0 | 1 | 2 | 5 | 10 | 20 | 30 |
| --- | --- | --- | --- | --- | --- | --- | --- |
| CK | A | B | B | B | B | B | A |
| 20E | A | A | A | A | A | A | A |
| 20E+Suramin | A | AB | AB | A | A | AB | A |
| *F* | 0.953 | 17.027 | 19.708 | 36.769 | 41.197 | 15.770 | 3.251 |
| *P* | 0.437 | 0.0059 | 0.0023 | 0.0004 | 0.0003 | 0.0041 | 0.1106 |

Note：Different letters indicate significant differences by Turkey test.
