# Supplementary material for: Calcineurin-dependent 20E signaling pathway enhances attacin expression to prime the immunity of Helicoverpa armigera against Bacillus thuringiensis Cry1Ac toxin
Source: PLoS Pathog. 2026 Aug 7;22(8):e1014503. doi: 10.1371/journal.ppat.1014503 (PMC13472496; doi:10.1371/journal.ppat.1014503)
Supplement: Supplementary file 5 [file ppat.1014503.s004.docx]

Table S4 Turkey test to compare significant differences by DPS7.05 for Figure 8b

| Sample | 0 | 5 | 10 | 20 | 30 |
| --- | --- | --- | --- | --- | --- |
| CK | A | B | B | C | C |
| 20E | A | A | A | A | A |
| 20E+Suramin | A | A | A | B | B |
| *F* | 0.7110 | 36.778 | 32.728 | 395.288 | 1467.647 |
| *P* | 0.5281 | 0.0004 | 0.0006 | 0.0001 | 0.0001 |

Note：Different letters indicate significant differences by Turkey test.
