# Supplementary material for: Calcineurin-dependent 20E signaling pathway enhances attacin expression to prime the immunity of Helicoverpa armigera against Bacillus thuringiensis Cry1Ac toxin
Source: PLoS Pathog. 2026 Aug 7;22(8):e1014503. doi: 10.1371/journal.ppat.1014503 (PMC13472496; doi:10.1371/journal.ppat.1014503)
Supplement: Supplementary file 6 [file ppat.1014503.s005.docx]

Table S5 Turkey test to compare significant differences by DPS7.05 for Figure 8c

| Sample | 0 | 1 | 2 | 5 | 10 | 20 | 30 |
| --- | --- | --- | --- | --- | --- | --- | --- |
| CK | A | A | B | B | B | A | A |
| 20E | A | A | A | A | A | A | A |
| 20E+Suramin | A | A | AB | AB | AB | A | A |
| *F* | 0.000 | 2.316 | 17.060 | 35.577 | 11.882 | 2.284 | 2.843 |
| *P* | 0.9999 | 0.1797 | 0.0033 | 0.0005 | 0.0082 | 0.183 | 0.1354 |

Note：Different letters indicate significant differences by Turkey test.
