# Supplementary material for: Calcineurin-dependent 20E signaling pathway enhances attacin expression to prime the immunity of Helicoverpa armigera against Bacillus thuringiensis Cry1Ac toxin
Source: PLoS Pathog. 2026 Aug 7;22(8):e1014503. doi: 10.1371/journal.ppat.1014503 (PMC13472496; doi:10.1371/journal.ppat.1014503)
Supplement: Supplementary file 8 [file ppat.1014503.s007.docx]

Table S7 Turkey test to compare significant differences by DPS7.05 for Figure 8g

|  | Sample | 0 | 0.5 | 1 | 2 | 3 |
| --- | --- | --- | --- | --- | --- | --- |
| CK | | A | B | C | C | B |
| 20E | | A | A | A | A | A |
| 20E+FK506 | | A | B | C | C | B |
| 20E+suramin | | A | B | B | B | B |
| *F* | | 1.091 | 45.145 | 199.878 | 466.396 | 27.311 |
| *P* | | 0.407 | 0.0001 | 0.0001 | 0.0001 | 0.0001 |

Note：Different letters indicate significant differences by Turkey test.
