# Supplementary material for: Calcineurin-dependent 20E signaling pathway enhances attacin expression to prime the immunity of Helicoverpa armigera against Bacillus thuringiensis Cry1Ac toxin
Source: PLoS Pathog. 2026 Aug 7;22(8):e1014503. doi: 10.1371/journal.ppat.1014503 (PMC13472496; doi:10.1371/journal.ppat.1014503)
Supplement: Supplementary file 9 [file ppat.1014503.s008.docx]

Table S8 Turkey test to compare significant differences by DPS7.05 for Figure 8h

| Sample | 0 | 0.5 | 1 | 2 | 3 |
| --- | --- | --- | --- | --- | --- |
| CK | A | B | B | A | A |
| 20E | A | A | A | A | A |
| 20E +FK506 | A | B | B | A | A |
| 20E +suramin | A | AB | B | A | A |
| *F* | 1.314 | 16.353 | 55.568 | 5.900 | 0.598 |
| *P* | 0.3356 | 0.0009 | 0.0001 | 0.0200 | 0.634 |

Note：Different letters indicate significant differences by Turkey test.
