# Supplementary material for: Calcineurin-dependent 20E signaling pathway enhances attacin expression to prime the immunity of Helicoverpa armigera against Bacillus thuringiensis Cry1Ac toxin
Source: PLoS Pathog. 2026 Aug 7;22(8):e1014503. doi: 10.1371/journal.ppat.1014503 (PMC13472496; doi:10.1371/journal.ppat.1014503)
Supplement: Supplementary file 10 [file ppat.1014503.s009.docx]

| Table S9 List of primers in this study | |
| --- | --- |
| Primer name | Sequence（5’-3’） |
| CDS-CaN-F | ATGTCCGGGAGCAATGATAAAG |
| CDS-CaN-R | CGAATGTGCGTTGCTGTTTACAT |
| qCaN-F | ATGAATGCCGACATTTGACA |
| qCaN-R | CCAAATGCAGGTGGTTCTTT |
| 30a-CaN-F | GCCATGGCTGATATCGGATCCATGTCCGGGAGCAATGATAAAG |
| 30a-CaN-R | TTGTCGACGGAGCTCGAATTCCGAATGTGCGTTGCTGTTTACAT |
| RFP-CaN-F | TCGTTAACACGTCAAGAGCTCATGTCCGGGAGCAATGATAAAG |
| RFP-CaN-R | TGCAGGCGCGCCGAGATCTGCGAATGTGCGTTGCTGTTTACAT |
| CDS-Dorsal-F | ATGGCGCGCCGCGACCA |
| CDS-Dorsal-R | GTAGTCGAGTGCGGGCTG |
| qDorsal-F | ATGTGAGTAATAAGAGGCGGAAGG |
| qDorsal-R | GGCGGACTGCTAATGTTATGGT |
| Dorsal-ORF-F | ATGGCGCGCCGCGACCA |
| Dorsal-ORF-R | GTAGTCGAGTGCGGGCTG |
| GFP-Dorsal-F | TCGTTAACACGTCAAGAGCTCATGGCGCGCCGCGACCA |
| GFP-Dorsal-R | TGCAGGCGCGCCGAGATCTGGTAGTCGAGTGCGGGCTG |
| RFP-Relish-F | TCGTTAACACGTCAAGAGCTCATGTCTACAAGCAGTGACCACGAT |
| RFP-Relish-R | TGCAGGCGCGCCGAGATCTGATCGATATACCTCCTTATGACACGT |
| qattacin-F | ATGTTCTCTTTAGTTTGCTTAACCCTG |
| qattacin-R | ATCGTCACCAGTTATTGGCAGTC |
| 30a-attacin-F | GCCATGGCTGATATCGGATCCATGTTCTCTTTAGTTTGCTTAACCC |
| 30a-attacin-R | TTGTCGACGGAGCTCGAATTCAAAATACTTTCTGAAAGTAAGACCA |
| RFP-attacin-F | TCGTTAACACGTCAAGAGCTCATGTTCTCTTTAGTTTGCTTAACCC |
| RFP-attacin -R | CCTGCAGGCGCGCCGAGATCTGAAAATACTTTCTGAAAGTAAGACCA |
| β-actin-F | CCTGGTATTGCTGACCGTATGC |
| β-actin-R | CTGTTGGAAGGTGGAGAGGGAA |
| EF-1α-F | GCCTGGTACCATTGTCGTCT |
| EF-1α-R | GTAACCACGACGCAACTCCT |
