# Supplementary material for: Calcineurin-dependent 20E signaling pathway enhances attacin expression to prime the immunity of Helicoverpa armigera against Bacillus thuringiensis Cry1Ac toxin
Source: PLoS Pathog. 2026 Aug 7;22(8):e1014503. doi: 10.1371/journal.ppat.1014503 (PMC13472496; doi:10.1371/journal.ppat.1014503)
Supplement: Supplementary file 11 [file ppat.1014503.s010.docx]

Figure S1 Expression of CaN protein and preparation of antibodies. (a) SDS-PAGE analysis of CaN protein expression induced by IPTG. M: protein marker;1: no induction; 2: proteins induced by IPTG; 3: protein located in the supernatant; 4: protein located in the pellet. (b) Western blot analysis to verify the expression of CaN protein. M: protein marker; 1: proteins induced by IPTG; 2: proteins not induced by IPTG.
